# Atg5^flox^-Derived Autophagy-Deficient Model of Pompe Disease: Does It Tell the Whole Story?

**DOI:** 10.1016/j.omtm.2017.08.002

**Published:** 2017-09-22

**Authors:** Jeong-A Lim, Hossein Zare, Rosa Puertollano, Nina Raben

**Affiliations:** 1 Laboratory of Muscle Stem Cells and Gene Regulation, National Institute of Arthritis and Musculoskeletal and Skin Diseases, National Institutes of Health, Bethesda, MD, USA; 2 Cell Biology and Physiology Center, National Heart, Lung, and Blood Institute, National Institutes of Health, Bethesda, MD, USA

## Main Text

Defective autophagy is a prominent feature in Pompe disease, an inherited deficiency of acid alpha-glucosidase. An absence of this enzyme leaves cells unable to digest glycogen in the lysosome. The major tissues affected by glycogen accumulation are cardiac and skeletal muscles. The currently available enzyme replacement therapy (ERT) works well in cardiac, but not in skeletal muscle. The disappointing response to therapy is linked to the presence of large areas of autophagic debris in muscle fibers. We have previously reported the generation of muscle-specific/autophagy-deficient Pompe mice by inactivating the Atg5 gene. To create this model, we used a mouse line that is employed by many other scientists—animals bearing an Atg5^flox^ allele in which exon 3 of the gene is flanked by lox*P* sequences. Unexpectedly, this genetic manipulation, instead of improving the disease phenotype, resulted in worsening of clinical manifestations. In this commentary, we present additional data suggesting that the negative effect of Atg5 inactivation in Pompe mice was associated with the generation of a potentially harmful product from the exon 3-floxed Atg5 allele.

In the process of studying the pathogenesis of Pompe disease, a severe metabolic myopathy caused by a deficiency of the glycogen-degrading lysosomal acid alpha-glucosidase (GAA), we documented the presence of large areas of autophagic debris in the diseased muscle fibers. The enlarged glycogen-filled lysosomes fail to fuse with autophagosomes and degrade their content. This sets up the conditions for the development of the massively disruptive autophagic buildup. These autophagic regions are located in the inter-myofibrillar space and contain multiple vesicles with clearly identifiable features: multivesicular bodies and single-membrane lysosomes, small and large double-membrane autophagosomes filled with polymorphic material and glycogen, and structures containing multiple concentric electron-dense membranes. Immunostaining of single muscle fibers from GAA-knockout (GAA-KO) mice with lysosomal and autophagosomal markers confirmed that these conglomerates, which are often located in the core of the fiber, span almost the full length of the fiber and occupy up to 40% of its volume. This “elephant in the room” interrupts muscle striation and greatly diminishes the efficacy of ERT—the only available treatment for Pompe disease. ERT appears unable to resolve the autophagic buildup, which continues to expand as the illness progresses.^1^ In addition, defective autophagy affects trafficking and processing of the therapeutic enzyme-recombinant human GAA (alglucosidase alfa, Myozyme, Sanofi Genzyme).^2^

To dissect the role of autophagic buildup and intralysosomal glycogen accumulation in muscle weakness and wasting in Pompe disease, we generated muscle-specific autophagy-deficient GAA-KO mice. These GAA-KO mice (referred to as HSAcre:Atg5 DKO^3^) contain a Cre recombinase transgene under the control of the human skeletal actin promoter (HSAcre) and an Atg5 gene, in which exon 3 is flanked by *loxP* sites.^4^ Because of the extensive genetic manipulation that was required to create these mice,^3^ we made a second line, MLCcre:Atg7 DKO, as a back-up. In this model, another critical autophagic gene, Atg7, is excised in muscle by the Cre recombinase driven by the myosin light chain1f (MLC) promoter.^5^ Accumulation of autophagic substrates and the absence of LC3-II in muscle from either line indicated the efficient suppression of autophagy.^3^, ^5^ Of note, in the absence of the disruptive autophagic buildup, ERT cleared lysosomal glycogen in muscle very efficiently in both strains.^5^ Unexpectedly, the phenotype of the two double knockout strains was very different. HSAcre:Atg5 DKO were much sicker than GAA-KO mice, whereas MLCcre:Atg7 DKO (referred to as Atg7 DKO), appeared to be no worse, if not better, than the GAA-KO.^3^, ^5^

**Figure 1:**
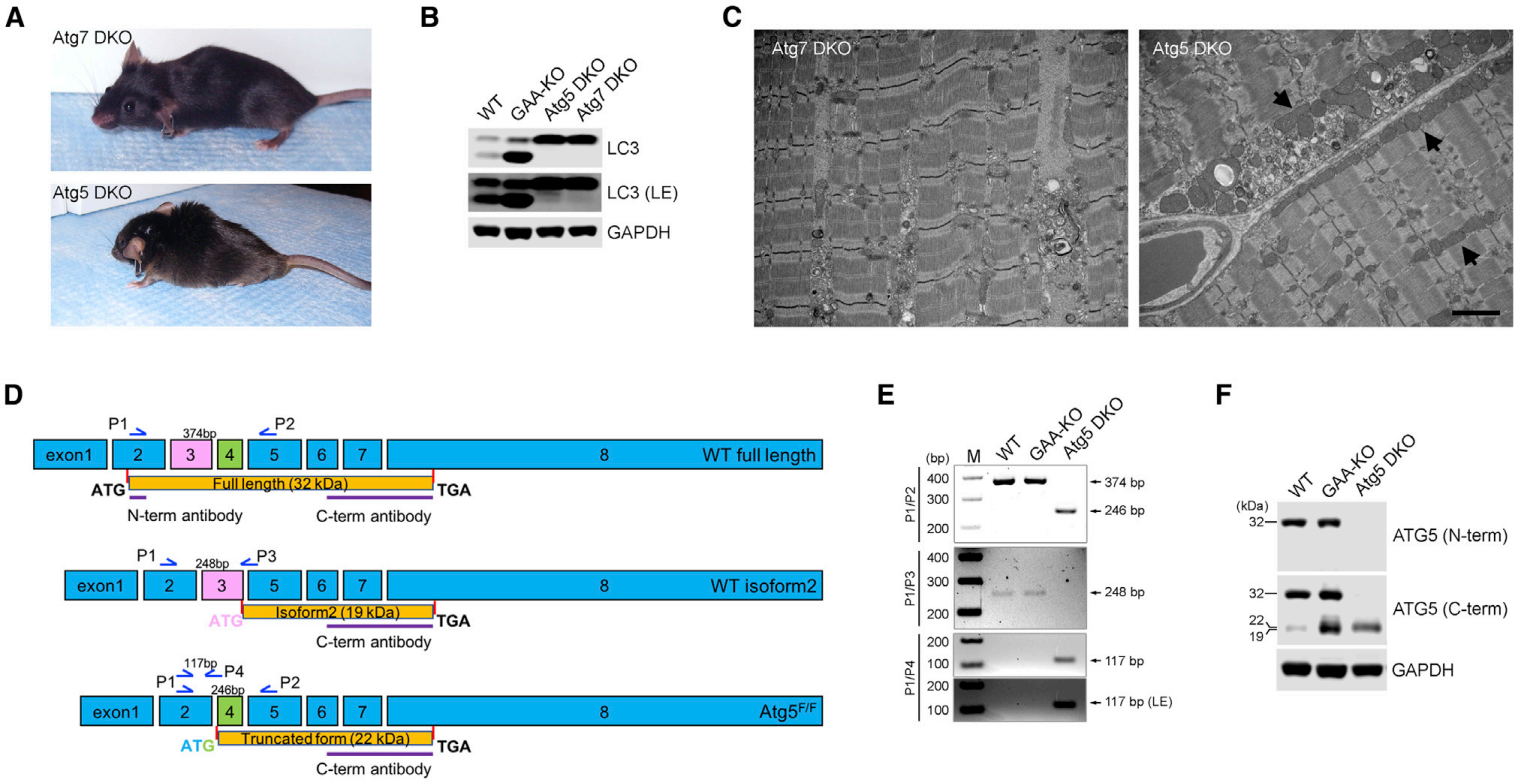
Exacerbation of Pompe Disease Phenotype in Atg5 DKO Is Associated with the Generation of a Potentially Toxic Product from the Transcript Lacking Exon 3 of the Atg5 Gene (A) The images show a severely affected 9-month-old Atg5/GAA DKO (Atg5 DKO), the only surviving male at this age; Atg7/GAA DKO (Atg7 DKO) mice exhibit much milder phenotype. (B) Western blot of protein lysates from muscles (gastrocnemius) derived from adult WT, GAA-KO, Atg5 DKO, and Atg7 DKO mice with LC3 antibody. The absence of LC3-II band in the samples from Atg5 DKO and Atg7 DKO confirms equally efficient suppression of autophagy in the double knockouts. LE, longer exposure. GAPDH was used as a loading control. (C) Electron-microscopy of muscle biopsies from 6-month-old Atg5 DKO and Atg7 DKO mice. Abnormal mitochondria are more prominent in Atg5 DKO muscle (arrows). Scale bar, 2 μm. (D) Splicing pattern of the alternatively spliced Atg5 transcripts is indicated. Isoform 2 is generated by splicing-out exon 4 of the gene. The truncated form is associated with exon 3-floxed Atg5 allele (Atg5^F/F^). The position of the primers used for PCR amplification is indicated. (E) RT-PCR analysis of muscle cDNA from WT, GAA-KO, and Atg5 DKO mice. The primers P1 and P2 detected 374 bp (full-length) amplification products in the WT and GAA-KO, but not in Atg5 DKO; similarly, a combination of primers P1 and P3 detected a 248 bp product in the WT and GAA-KO, but not in Atg5 DKO. The primers P1 and P2 detected a 246 bp product only in the Atg5 DKO muscle. The primers P1 and P4 detected a 117 bp product corresponding to the exon 3-spliced Atg5 transcript only in Atg5 DKO. M, DNA marker. (F) Western blot of protein lysates from muscles derived from WT, GAA-KO, and Atg5 DKO mice. The blot was probed with N-terminal or C-terminal anti-Atg5 antibodies. GAPDH was used as a loading control.

The reason for this difference was not clear. Both Atg5 and Atg7 function at the initial steps of autophagosomal formation,^6^ and inactivation of either of the genes resulted in suppression of autophagy in the skeletal muscle of GAA-KO mice.^3^, ^5^ To exclude a possibility of unforeseen genetic manipulation and/or different genetic background, we generated MLCcre:Atg5 DKO (referred to as Atg5 DKO), but the outcome was the same as with HSAcre:Atg5 DKO;^3^ at the age of 2–3 months, the animals showed obvious signs of muscle wasting and kyphosis, and, by the age of 6–7 months, they breathed with difficulty and developed a near paralysis of hind limbs. At this stage, these mice require daily observation and many begin to die (Figure 1A). These changes correlated with muscle atrophy and weight loss. In contrast, our observational data have shown that both GAA-KO and Atg7 DKO have similar mobility and lifespans (up to two years), and both develop the first clinical signs of muscle wasting by 8–9 months of age. Furthermore, the fibers isolated from Atg7 DKO generated a higher force than the GAA-KO fibers (our unpublished data). This time, the genetic background could not possibly account for the phenotypic difference because both Atg5 DKO and Atg7 DKO were on the same background and each strain was maintained by sibling mating for several generations. We have also compared the levels of LC3-I/LC3-II—the key autophagic protein commonly used to monitor autophagy^7^—in the two DKO strains and did not detect any difference between them (Figure 1B).

Both GAA-KO and Atg7 DKO are widely used to analyze the disease pathogenesis and the effect of different therapies. Thus, in practical terms, there was no need to use Atg5 DKO for the Pompe-related studies. However, driven by scientific curiosity, we compared electron micrographs from the two DKO strains. Large autophagic buildup that is prominent in GAA-KO muscle was not seen in double knockouts, although small clusters of autophagy-related vesicles and aberrant mitochondria were detected in both strains. Strikingly enlarged abnormal mitochondria in the intermyofibrillar and subsarcolemmal regions were more prominent in muscle from Atg5 DKO compared to Atg7 DKO—perhaps, the only noticeable difference between the two strains by electron microscopy (EM) (Figure 1C).

Next, we performed high-throughput sequencing of mRNA (RNA sequencing [RNA-seq]) using muscle from the two DKO strains (n = 4 for Atg5 DKO and n = 6 for Atg7 DKO). As expected, the expression level of Atg7 was high in Atg5 DKO and barely above the threshold value in Atg7 DKO. In contrast, the expression levels of Atg5 were high in both strains except for exon 3 of the gene in Atg5 DKO; the number of reads for exon 3 was below the threshold value (Figure S1). The RNA-seq-derived Atg5 cDNA sequence contained 1,240 bp and included all exons except exon 3. The splicing out of exon 3 creates a new ATG start codon at the junction of exon 2 and 4 (3′ end of exon 2 …CATACT**AT/G**CATTAT……5′ end of exon 4; the underlined sequence surrounding the new start site satisfies the Kozak rule). These data suggested that the alternatively spliced transcript (without exon 3) generated from the exon 3-floxed Atg5 allele may encode a product (22 kDa) that is toxic to the cells.

We then analyzed differentially expressed genes in Atg5 DKO and Atg7 DKO and compared them to the previously reported mRNA-seq data for GAA-KO and WT muscle samples (GEO: GSE57980).^8^ Differential expression was selected based on fold changes (≥2.0) and the reproducibility among replicates of the same condition. This allowed us to identify a group of genes that were upregulated (≥2-fold; p < 0.05) in Atg5 DKO relative to the other three datasets: Atg7 DKO, GAA-KO, and wild-type (WT). Gene ontology (GO) enrichment analysis of these genes returned a small but highly significantly upregulated set of genes encoding proteins involved in detoxification response (Table S1).

Several alternatively spliced transcript variants for the Atg5 gene have been annotated by the NCBI Reference Sequence Database (Ref-Seq), including, but not limited to, variants with deletion of exon 3, exon 4, exon 6, and a combination of exons 3 and 6. A transcript variant lacking exon 3 of the human Atg5 has been documented in the database twice (NCBI: NM_001286107.1). In both cases, fetal brain tissue specimens were used to generate the Atg5 cDNA, and the predicted encoded isoform of 197 amino acids is similar to the one generated from the exon 3-floxed Atg5 allele. Two transcript variants (detected in DU145 prostate cancer cells) lacking exon 6 or a combination of exons 6 and 3 were reported in the literature^9^ and documented in the database (JQ918353 and JQ918354, respectively). The only Ref-Seq annotated transcript variant for mouse Atg5 (variant 2; NM_001314013.1) is the one lacking exon 4 of the gene. This variant initiates translation at a start codon at the 3′ end of exon 3, and the predicted protein (Atg5 isoform 2) of 19 kDa has a different N terminus compared to the full-length Atg5 protein of 32 kDa (Figure 1D). The function of this transcript variant or its product remains unknown.

RT-PCR with primers (see Figure 1D for the position of the primers and Supplemental Information for the sequences) in exon 2 (P1) and exon 5 (P2) of the Atg5 gene detected WT product (374 bp) in the WT and GAA-KO, but not in Atg5 DKO. RT-PCR with primers P1 and P3 (at the exons 3/5 junction) yielded a product of 248 bp, which corresponds to the exon 4-deleted Atg5 isoform 2 transcript. Again, this product was seen in the WT and GAA-KO, but not in Atg5 DKO. A 246 bp product amplified with P1 and P2, which corresponds to exon 3-spliced transcript, was seen only in Atg5 DKO (Figure 1E). We have also considered a possibility that a small amount of the exon 3-spliced Atg5 transcript can be normally generated in mouse tissues since this transcript has been reported in humans (see above). Therefore, we used P1 and antisense primer P4 positioned at the exons 2/4 junction. However, the expected product of 117 bp was detected only in Atg5 DKO, but not in the WT or GAA-KO, thus arguing against such a possibility (Figure 1E).

Next, we performed western blot analysis of muscle lysates from WT, GAA-KO, and Atg5 DKO mice. As expected, the antibody that recognizes the N terminus of ATG5 (Abgent; #AP1812a) detects the full-length protein in WT and GAA-KO, but not in Atg5 DKO (Figure 1F). In both WT and GAA-KO, the antibody that recognizes the C terminus (Abnova; #H00009474-M04) detects the full-length protein and a smaller product (19 kDa), which likely corresponds to Atg5 isoform 2 lacking exon 4. A slightly higher (22 kDa) strong band detected with the C-terminal antibody in Atg5 DKO appears to correspond to the truncated form generated from the exon 3-floxed Atg5 allele. Taken together, these data suggest that the generation of the Atg5-truncated fragment may aggravate the phenotype of the acid alpha-glucosidase-deficient mice, thus explaining the difference between the Atg5 and Atg7 double knockouts.

In conclusion, the purpose of this commentary is 2-fold. (1) Our published data on the exacerbation of the Pompe disease phenotype in HSAcre:Atg5 DKO^3^ called into question a net benefit of inhibition of autophagy upstream of autophagosome formation and led to speculation over the possible protective role of autophagosomal accumulation in Pompe skeletal muscle.^10^, ^11^ The results presented here can put these thoughts to rest. (2) We would like to alert any other researchers who use mice carrying an Atg5^flox^ allele in which exon 3 of the Atg5 gene is flanked by lox*P* sites. It is certainly possible that the impact of the Atg5-truncated product can be mitigated in non-muscle tissues or in the context of normal lysosomes. Furthermore, we would like to emphasize that the final verdict on the presence and functional significance of the truncated Atg5 form awaits further experimental evidence, such as sequencing of the protein band detected with the C-terminal Atg5 antibody, expression of the truncated product in Atg5 null cells, generation of transgenic mice expressing the product, etc. However, one thing is certain: the removal of exon 3 creates an ATG and, at least in muscle, the exon 3-deleted Atg5 mRNA is highly expressed.

Finally, while pursuing this Pompe disease “side project,” we have noticed that the abundance of exon 4 spliced-out Atg5 isoform 2 in GAA-KO muscle is much higher than in the wild-type (Figure 1E). This finding brings us back to Pompe research. The functional significance of the enhanced levels of Atg5 isoform 2 in the diseased muscle awaits further investigation. As we are often reminded, the path ahead has never been straightforward.

## Author Contributions

J.L. performed experiments, analyzed and interpreted the data, and participated in preparation of the manuscript; H.Z. analyzed and interpreted the RNA-seq data; R.P. analyzed and interpreted the data and participated in writing of the manuscript; N.R. generated mouse models, designed, interpreted, and analyzed data, and wrote the paper.

## Conflicts of Interest

The authors declare no conflict of interest.

## Supplemental Information

Document S1. Supplemental Materials and Methods, Figure S1, and Table S1

Document S2. Article plus Supplemental Information
