# Supplementary material for: Atg5flox-Derived Autophagy-Deficient Model of Pompe Disease: Does It Tell the Whole Story?
Source: Mol Ther Methods Clin Dev. 2017 Sep 22;7:11–4. doi: 10.1016/j.omtm.2017.08.002 (PMC5640697; doi:10.1016/j.omtm.2017.08.002)
Supplement: Document S1. Supplemental Materials and Methods, Figure S1, and Table S1 [file mmc1.pdf]

OMTM, Volume *7*

Supplemental Information

Atg5^{flox}-Derived Autophagy-Deficient Model of Pompe Disease: Does It Tell the Whole Story?

Jeong-A Lim, Hossein Zare, Rosa Puertollano, and Nina Raben

Supplemental Information

	Atg5 DKO (RPKM)				Atg7 DKO (RPKM)						Average (RPKM)			FC
Gene	1	2	3	4	1	2	3	4	5	6	Atg5 DKO	Atg7 DKO	p	(Atg7DKO/Atg5DKO)
Atg5	11.46	10.56	10.71	8.49	11.58	10.63	10.67	9.41	13.76	13.66	10.31	11.62	0.2397	1.13
Atg7	4.28	4.01	4.01	4.61	0.51	0.52	0.40	0.61	0.75	0.51	4.23	0.55	2.36561E-09	0.15

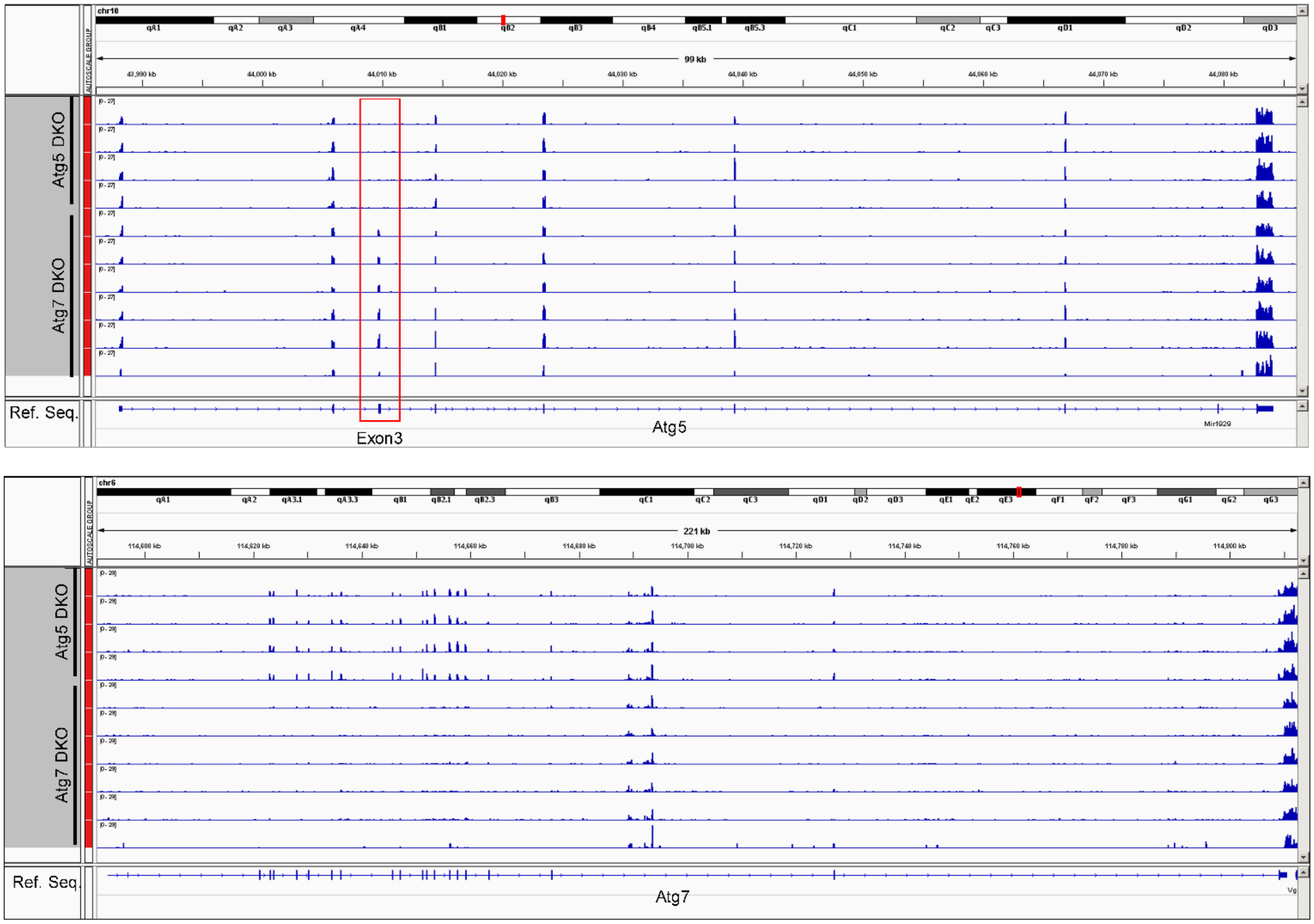

Figure S1. Visualization of RNA-seq coverage across the Atg5 (top) and Atg7 (bottom) genes. Expressed exons are seen as peaks, and are consistent with RefSeq mouse genome annotation. Note lack of reads corresponding to exon 3 of the Atg5 gene in all 4 samples from Atg5 DKO. The values on the top table for Atg5 and Atg7 represent RPKM (Reads Per Kilobase of transcript per Million mapped reads). The raw sequencing datasets are available upon request.

Table S1. GO terms for the most significantly upregulated genes in muscle from Atg5 DKO (≥ 2-fold) compared to WT, GAA-KO, and Atg7 DKO.

Gene	Average (RPKM)		p-value	FC (Atg5DKO/Atg7DKO)
	Atg5 DKO	Atg7 DKO		
Mir3101	59.84	0.00	0.000691593	N/A
Arhgap21	3.34	0.00	8.77409E-11	N/A
Hal	1.50	0.03	5.76272E-06	54.61
Chia	1.14	0.02	0.000324877	46.25
Abhd1	3.35	0.28	8.57784E-05	12.04
Flywch2	3.38	0.30	0.000257637	11.45
Golm1	5.34	0.52	0.009501052	10.20
Park2	21.85	4.50	2.14812E-07	4.86
BC048679	12.04	2.64	0.00442623	4.55
Nnat	6.42	1.49	0.000262458	4.31
Fibin	17.38	5.26	0.000217099	3.30
Asns	44.52	13.95	8.18065E-05	3.19
Fos	11.38	4.44	0.017088314	2.56
Wnt5b	3.31	1.33	7.09606E-05	2.48
Car4	4.50	1.92	0.008870785	2.35
Acot2	36.24	16.18	0.03327828	2.24
Apold1	3.01	1.40	0.014157153	2.15
Nt5dc2	1.20	0.53	0.004442646	2.27
Dysf	32.00	15.59	9.8555E-06	2.05
Slc25a34	14.46	7.12	0.023259494	2.03

Category	Term	Description	LogP	Symbols
GO Biological Processes	GO:0009636	response to toxic substance	-3.1985	ASNS,FOS,PRKN
GO Biological Processes	GO:0050708	regulation of protein secretion	-2.5035	NNAT,PRKN,CHIA
GO Biological Processes	GO:0032787	monocarboxylic acid metabolic process	-2.0432	ASNS,HAL,ACOT2
GO Biological Processes	GO:0010256	endomembrane system organization	-2.0180	PRKN,DYSF,ARHGAP21

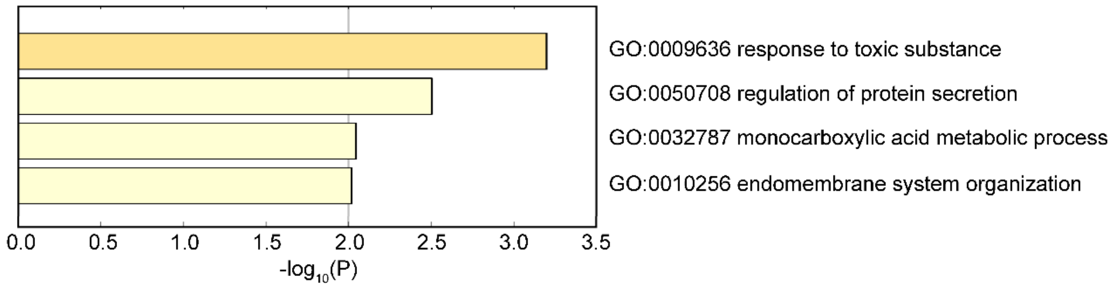

Biological Process Ontology highlighted a set of genes which are upregulated in response to toxic substances. GO analysis was performed with Metascape 1.0 (http://metascape.org) (P-value <0.01).

List of primers for the RT-PCR amplification:

P1 (Atg5 exon 2s): GATGACAAAGATGTGCTTCGAGAT

P2 (Atg5 exon 5as): GACATAAAGTGAGCCTCAACCGCAT

P3 (Atg5 exon3/5 junction-as): GTCCTTTTCTGGAAAACTCATTTCA

P4 (Atg5 exon2/4 junction – as): CCAATTGGATAATGCATAGTAT
